# The Evolving Role of Bridging Therapy during CAR-T Therapy

**DOI:** 10.46989/001c.116261

**Published:** 2024-07-16

**Authors:** Shakthi T Bhaskar, Bhagirathbhai Dholaria, Bipin N Savani, Olalekan Oluwole

**Affiliations:** 1 Medicine, Hematology and Oncology Vanderbilt University Medical Center; 2 Chimeric antigen receptor Vanderbilt University Medical Center

**Keywords:** Chimeric antigen receptor, bridging therapy

## Abstract

Chimeric antigen receptor (CAR) T-cell therapy has gained wide used across an array of hematologic malignancies. Though CAR T therapy has changed outcomes in the treatment of many malignancies its administration can be complicated by delays related to patient-related factors, social barriers, or insurance issues. Because of the lengthy process required to treat a patient with CAR T-cells, bridging therapy (BT), administered after leukapheresis but prior to CAR T infusion, has become an important component of safely administering CAR T therapy. Here we review data supporting the use of BT, factors to consider in patient selection, and types of available BT and rationale for choosing amongst them.

## Introduction

Chimeric antigen receptor (CAR) T-cell products have been approved for use in several malignancies, including large B-cell lymphoma (LBCL), B-cell precursor acute lymphoblastic leukemia (B-ALL), follicular lymphoma (FL), mantle cell lymphoma (MCL), and multiple myeloma (MM). The available products vary in label indication, line of therapy, and side effect profile. However, the steps patients undergo to receive CAR T therapy is similar across the various product types, and includes initial evaluation, leukapheresis, manufacturing, lymphodepletion, and, finally, CAR T infusion.

The process of getting a patient to CAR T treatment is complicated and can include delays at various steps, including obtaining insurance authorization and during cell manufacturing. Because of this, bridging therapy (BT) is often used to stabilize patients’ underlying malignancy while awaiting CAR T infusion.

As CAR T therapy has evolved over time, so has the use of BT. In this review, we provide a summary of the evidence for use of BT, guidance for patient selection, and discuss various options for the type of BT that can be used.

## Evidence for the use of BT

BT is local or systemic treatment that is delivered after leukapheresis for CAR T-cells has been completed, and before lymphodepleting chemotherapy and CAR T-cell infusion. The goal of BT is to rapidly stabilize progressive disease while awaiting more definitive treatment with CAR T-cells. In addition to true BT, many patients receive post-referral therapy, which is local or systemic treatment that is delivered after the patient has been referred for CAR T-cell therapy, but while their evaluation and insurance authorization is underway prior to leukapheresis.

There was significant variation in the use of BT amongst the major clinical trials which led to the approval of different CAR T products (Table 1). For example, BT was not allowed in ZUMA-1, which evaluated axicabtagene ciloleucel (axi-cel, Yescarta) in LBCL.^1^ The subsequent ZUMA-7 study, comparing axi-cel to autologous stem cell transplantation (ASCT) as second line therapy, allowed BT in the form of glucocorticoids only. In that study, 65 patients (36%) in the axi-cel arm received BT. The ZUMA-5 study, which led to the approval of axi-cel in patients with follicular lymphoma (FL), allowed for the use of BT, but it was only administered to 6 of 148 patients.^2^

**Table 1.** 

**Study/Product**	**Disease**	**Total No. of Patients Enrolled/infused**	**No. of Patients Receiving Bridging (%)**	**Type of Bridging Therapy (No. of patients receiving)**	**CR/ORR**	**OS**	**Comments**
ELIANA (Tisagenlecleucel, Kymriah^©^) (NCT02435849)	B cell ALL	92/75	65 (87)	Not specified	60%/81%	76% at 12 months	Response to BT not available
ZUMA-3 (Brexucabtagene autoleucel, Tecartus^©^) (NCT02614066)	B cell ALL	71/55	51 (93)	Combinations of dexamethasone, vincristine, doxorubicin, mercaptopurine, hydroxyurea, methotrexate, fludarabine, cytarabine, idarubicin, cyclophosphamide, +/-tyrosine kinase inhibitor	71% CR or CRi, ORR not reported	71% at 12 months	34 (62%) patients had >25% bone marrow blasts after BT; 5 (9%) had <5% bone marrow blasts after BT
ZUMA-2 (Brexucabtagene autoleucel, Tecartus^©^) (NCT02601313)	MCL	74/68	25 (37)	Steroids (14), ibrutinib (14), acalabrutinib (5), or combination (6)	59%/85%	83% at 12 months	17 patients with imaging before and after BT – majority had PD; 3 patients died of PD before treatment
ZUMA-5 (Axicabtagene ciloleucel, Yescarta^©^) (NCT03105336)	Indolent lymphoma	153/146	6 (4)	Combinations of steroids (4), rituximab (4), bendamustine (2), etoposide (3), ifosfamide (3), carboplatin (2), mitoxantrone (1), cyclophosphamide (1), fludarabine (1), ibrutinib (1)	74%/92%	87.4% at 18 months	All patients had measurable disease after BT
ELARA (Tisagenlecleucel, Kymriah^©^) (NCT03568461)	Follicular lymphoma	98/97	44 (45)	Rituximab (10), steroids (8), gemcitabine (4), oxaliplatin (3), etoposide (3), cyclophosphamide (2), vincristine (2), bendamustine (1), radiation (1)	69.1%/86.2%	Median OS not reached	1 patient withdrawn due to CR to BT, all others had residual disease after BT
ZUMA-1 (Axicabtagene ciloleucel, Yescarta^©^) (NCT02348216)	Large B cell lymphoma	111/101	0 (0)	Not allowed	54%/82%	59% at 12 months	1 patient was not treated due to PD.
ZUMA-7 (Axicabtagene ciloleucel, Yescarta^©^) (NCT03391466)	Large B cell lymphoma	359/170 of 180 pts in axi-cel arm were infused	65 (36)	Steroids only (36)	65%/83%	61% at 2 years	Response to BT not available
JULIET (Tisagenlecleucel, Kymriah^©^) (NCT02445248)	Large B cell lymphoma	165/111	Not reported (92)	Combinations of rituximab, gemcitabine, etoposide, steroids, cisplatin, cytarabine, ibrutinib, lenalidomide	40%/52%	40% at 12 months	Response to BT not available
BELINDA (Tisagenlecleucel, Kymriah^©^) (NCT03570892)	Large B cell lymphoma	332/155 of 162 pts in tisa-cel arm were infused	135 (83.3)	Investigators choice of R-ICE, R-DHAP, R-GDP, R-GemOx	28.4%/46.3%	Not available	Response to BT not available
TRANSCEND (Lisocabtagene maraleucel, Breyanzi^©^) (NCT02631044)	Large B cell lymphoma	344/269	159 (59)	Combinations of rituximab, gemcitabine, oxaliplatin, steroids, bendamustine, lenalidomide, brentuximab vedotin, ibrutinib	53%/73%	57.9% at 12 months	BT did not result in lower tumor burden in most patients.
TRANSFORM (Lisocabtagene maraleucel, Breyanzi^©^) (NCT03575351)	Large B cell lymphoma	184/90 of 92 in liso-cel arm were infused	58 (63)	Investigators choice of one cycle of R-ICE, R-DHAP, or R-GDP	66%/86%	79.1% at 12 months	9 patients had a negative PET after BT and before liso-cel infusion but no difference in overall outcomes
KarMMa (Idecabtagene vicleucel, Abecma^©^) (NCT03361748)	Multiple myeloma	140/128	112 (88)	Dexamethasone, cyclophosphamide, daratumumab, carfilzomib, bortezomib, pomalidomide	33%/73%	78% at 12 months	5/112 (4%) patients who received BT responded; no complete responses were reported.
CARTITUDE (Ciltacabtagene autoleucel, Carvykti^©^) (NCT03548207)	Multiple myeloma	113/97	73 (75)	Not specified	67%/97%	89% at 12 months	Response to BT not available

CR: complete response rate; Cri: complete response with incomplete hematologic recovery rate; ORR: overall response rate; OS: overall survival, BT: bridging therapy; ALL: acute lymphoblastic leukemia; MCL: mantle cell lymphoma

In contrast, the ELIANA trial, which led to the approval of tisagenlecleucel (tisa-cel, Kymriah) for patients with B-cell ALL, 65 of the total 92 patients who were enrolled received BT, but details of regimens used are not available.^3^ The JULIET trial, which evaluated tisa-cel in LBCL, allowed the use of BT.^4^ The choice of therapy was diverse, including combinations of rituximab, gemcitabine, etoposide, steroids, cisplatin, cytarabine, ibrutinib, and lenalidomide. In the BELINDA study, which evaluated the use of tisa-cel in LBCL in the second line compared to autologous stem cell transplant (ASCT) BT was allowed in the tisa-cel group.^5^ In that study, 135 of the 162 patients on the tisa-cel arm received BT with the investigators’ choice of four platinum-based regimens. Patients who did not receive BT tended to be older, had refractory disease, a lower international prognostic index (IPI) score, and tended to have a disease other than LBCL. Patients outside of the United States tended to receive more BT than those in the United States. In the ELARA study, which evaluated the efficacy of tisa-cel in FL, BT was administered in 44 of 97 (45%) of patients, and consisted of combinations of rituximab, dexamethasone, gemcitabine, oxaliplatin, prednisolone, etoposide, cyclophosphamide, vincristine, and bendamustine. Radiation was used as BT in two patients.

The TRANSCEND study, which evaluated the use of lisocabtagene maraleucel (liso-cel, Breyanzi) in LBCL, enrolled 344 patients.^6^ Of these, 159 (59%) received BT, which included combinations of rituximab, gemcitabine, oxaliplatin, steroids, bendamustine, lenalidomide, brentuximab vedotin, and ibrutinib. The subsequent TRANSFORM study compared liso-cel to ASCT in the second line and allowed patients to receive one cycle of BT that consisted of one of the three chemoimmunotherapy regimens that were given to the ASCT arm.^7^ Among the 92 patients on the liso-cel arm, 58 (63%) were treated with BT, and 5 (9%) received more than one cycle.

The ZUMA-2 trial evaluating brexucabtagene autoleucel (brexu-cel, Tecartus) in MCL included 74 patients, 25 (35%) of whom received BT.^8^ Regimens for these patients included steroids, ibrutinib, acalabrutinib, or a combination.

The KarMMa trial, which evaluated the use of idecabtagene vicleucel (ide-cel, Abecma) in MM, enrolled 178 patients, of whom 112 (88%) received BT,^9^ consisting of dexamethasone, cyclophosphamide, daratumumab, carfilzomib, bortezomib, and pomalidomide. The KarMMa-3 trial, which evaluated ide-cel in patients who were refractory to between two and four prior lines of therapy, BT was allowed but details of this are not available.^10^

In the CARTITUDE-1 study evaluating the use of ciltacabtagene autoleucel (cilta-cel, Carvykti) in MM^11^ 73 of 97 (75%) patients received BT, which included corticosteroids, proteasome inhibitors, immunomodulatory agents, daratumumab, venetoclax, cisplatin, cyclophosphamide, melphalan, bendamustine, etoposide, and doxorubicin. In the CARTITUDE-3 study evaluating cilta-cel in patients with lenalidomide refractory MM, details of BT were not reported.^11^

There are also data available regarding patterns of use of BT in the real-world setting. One study published by the US Lymphoma CAR T Consortium evaluated the use of axi-cel for DLBCL in 298 patients across 17 centers.^12^ BT was used in 158 (53%) of patients and included combinations of steroids, chemotherapy, radiation, or targeted therapies such as lenalidomide or ibrutinib. In a multivariate analysis, patients who received BT had worse overall survival at twelve months (56% versus 81% in patients who did not receive BT, p<0.001). Another study evaluated the relationship between BT and outcomes in 148 patients with DLBCL who were treated with axi-cel.^13^ Eighty-one (55%) received BT which included systemic therapy, radiation therapy, or combined-modality therapy. Those patients who were treated with BT tended to have an elevated IPI, bulky disease, and an elevated lactate dehydrogenase (LDH). The one year overall survival (OS) was also worse in patients who received BT, reported at 48% (versus 65% in patients who did not receive bridging, p=0.05).

In a retrospective analysis, 46 patients who received commercial CAR T products were evaluated.^14^ Thirty (65%) were treated with high-intensity BT, defined as chemotherapy with or without immunotherapy, and 16 (35%) received low intensity or no BT. The intensity of BT was closely related to tumor burden at enrollment and, while there was no difference in efficacy of CAR T between the groups, the high-intensity group did have a higher frequency of cytokine release syndrome and neurotoxicity. Another single-institution retrospective study evaluated 64 patients with non-Hodgkin lymphoma, 49 of whom received commercial CAR T.^15^ In 34 (69%) the purpose of BT was to reduce tumor burden or palliate symptoms. BT included combination chemoimmunotherapy, radiation alone, systemic therapy with radiation therapy, targeted treatments, or combination treatment.

Finally, a study from the United Kingdom evaluated 375 patients with relapsed/refractory LBCL who underwent leukapheresis for CAR T. Of these patients, 326 (87%) received BT, in the majority consisting of a single cycle of chemotherapy.^16^ Twenty-three patients achieved a complete response (CR) after BT, with those with a CR or PR showing an improved one-year OS of 63.2% after CAR T versus 45.9% in patients with no response to BT (p=0.001). This relationship was consistent in a multivariate analysis which showed a reduction in the risk of disease progression or death in patients who achieved a CR or PR to BT.

## Patient Selection

The data presented above underscore the importance of careful patient selection. Patients who receive BT are predominantly those with a higher disease burden and rapidly progressive disease. These patients tend to have worse overall outcomes, likely related to their aggressive underlying disease. They also tend to have more severe toxicities with CAR T-cells as high LDH, bulky disease, and higher total metabolic tumor volume have all been associated with development of more severe cytokine release syndrome (CRS) and immune effector cell-associated neurotoxicity syndrome (ICANS).

However, there are data to imply that patients who respond to BT may have improved outcomes relative to those who do not, which may shed additional light on optimal patients to undergo BT and subsequent CAR T-cell therapy. As discussed below, BT regimens must be carefully selected with an emphasis on limiting toxicity, but individualizing selection of these regimens to what will help a patient achieve an optimal response is also critical. Further research is needed to know whether the goal of BT should not simply be to stabilize disease but, rather, to achieve an objective response when able.

BT should also be considered for patients in whom delays in receiving CAR T treatment are expected, whether for social or financial reasons. These patients often receive post-referral therapy while undergoing CAR T evaluation or awaiting leukapheresis, which may eliminate the need for BT after leukapheresis. Very little data are available on the types of post-referral therapy regimens or impact of these treatments on patient outcomes.

It is important to recognize that not all patients require BT prior to CAR T treatment. Avoiding BT where not needed may help to preserve performance status and mitigate cost of treatment. Patients who may be considered for observation alone include those with slowly progressive disease who are otherwise clinically stable and minimally symptomatic. These patients should be monitored at regular intervals to ensure indications for BT do not develop while awaiting CAR T treatment. Additionally, limited data are available regarding BT in real-world patients with MM being treated with B cell maturation antigen (BCMA)-targeted CAR T-cells.

## Type of Bridging

### Chemoimmunotherapy

Combination chemoimmunotherapy has typically been the form of BT that is used most often. There are few studies that report details of regimens used in LBCL (and no data available in MM). When using chemoimmunotherapy as BT, a few important aspects of treatment should be considered.

Previously used regimens should be avoided, and any residual side effects from prior treatment should be considered when choosing a BT regimen. Those that may cause significant or long-standing myelosuppression should be avoided when possible, particularly as there is no evidence that more intensive chemotherapy improves response rates or outcomes with CAR T therapy. Regimens that cause significant lymphopenia should not be used, given the importance of lymphodepleting chemotherapy prior to CAR T infusion. Infectious risk should also be carefully considered.

### Targeted Therapy

Targeted medications such as immunomodulatory agents (IMIDs), Bruton’s tyroskine kinase (BTK) inhibitors, proteasome inhibitors, venetoclax, blinatumomab, and inotuzumab ozogamicin have all been used as BT. There are limited data to guide selection of these agents, but similar safety and side effect profile evaluations should take place as with chemoimmunotherapy.

One important potential pitfall is to avoid the use of agents that recognize the same antigenic target as the chosen CAR T product, though emerging data may shift away from this practice in the future. In the Zuma-3 study that evaluated the use of brexu-cel in patients with relapsed/refractory B-ALL, those who received prior blinatumomab treatment were included if leukemic blasts continued to have >90% CD19 expression.^17^ In a subgroup analysis, relapse-free and OS rates were similar between patients who did and did not receive prior blinatumomab. Rates of CD19 expression loss were note reported in that analysis.

In LBCL, recent approvals of CD19-targeted agents such as tafasitamab and loncastuximab terisine have raised theoretical concerns about loss of CD19 expression and subsequent CAR T therapy. However, in an analysis of 12 patients’ lymph node samples from the L-MIND study, which led to the approval of tafasitamab in combination with lenalidomide in patients with relapsed/refractory LBCL, CD19 expression, intensity, and distribution were retained in patients after treatment with a median of 14.5 doses.^18^ Similarly, pooled results from clinical trials including 14 patients who received CD19-directed CAR T cell therapy after treatment with loncastuximab terisine showed that half of them responded to CAR T-cells with 6/14 achieving a CR.^19^

When considering the clinical application of these data, it should be highlighted that these studies included very small sample sizes, and further investigation is needed to assess the efficacy of CAR T cell therapy after these targeted treatments.

### Radiation

Radiation can also be considered as BT, particularly in patients with limited sites of disease or patients who are at risk for structural complications such as airway compromise or renal dysfunction. In one study, radiation therapy (RT) alone was shown to be a safe and effective bridging strategy in a small cohort (n=11).^13^ Patients who were bridged with RT had similar rates of toxicity as those who received systemic or no bridging, and had an improved progression-free survival compared to patients who were bridged with systemic therapy. In another small study, 12 patients received RT as bridging before receiving axi-cel. RT was shown to be safe, and the response and complication rates after axi-cel were similar to those in patients who received other forms of BT or no BT.^20^

One CAR T therapy study in MM included four patients who received RT as bridging.^21^ These patients did not have increased rates of radiation- or CAR T-associated toxicities, and their progression-free survival (PFS) and OS were similar to those who did not receive RT as bridging. This provides some rationale for use of RT as bridging in MM as well, though further studies are needed.

The role of BT for patients undergoing CAR T cell treatment has evolved over time, but BT remains an important tool for many patients. Outcomes with BT have varied between clinical trials and real-world studies. For example, the Zuma-1 study did not allow BT but demonstrated excellent response rates and outcomes, while commercial implementation of axi-cel has necessitated the use of BT in many patients. These discrepancies are likely related to the variability in time to leukapheresis and manufacturing time between trials and in real-world experience, where the CAR T-cell process is often limited by delays related to disease status, social barriers, or insurance authorization.

Since detailed data to provide guidance when using BT are still limited, clinical judgement is critical when evaluating whether a patient needs BT, what the goal of BT administration is, and what type of BT is optimal for a given patient. With axi-cel and liso-cel now available for patients as second-line therapy, clinical judgment is also needed to determine how to manage patients who have a CR after receiving BT, and whether they are best served by proceeding with CAR T or should be considered to have chemotherapy-sensitive disease and moved to ASCT instead.

Future studies in this area should focus on identifying what types of BT regimens lead to the best outcomes for patients after CAR T treatment, whether stable disease is an optimal goal of BT administration, whether targeted therapies can safely be administered as BT, and what the optimal bridging strategies in MM are. As CAR T therapy is increasingly used in the second line, future studies should be conducted to guide decisions about CAR T or ASCT in patients who respond well to BT.

### Authors contribution per CRediT

Conceptualization All authors

Data curation

Formal Analysis

Funding acquisition

Investigation

Methodology All authors

Project administration

Resources

Software

Supervision Oluwole

Validation

Visualization

Writing – original draft Bhaskar, Oluwole

Writing – review & editing All authors

### Corresponding Author

Shakthi T. Bhaskar

Vanderbilt University Medical Center

2220 Pierce Avenue, 777 PRB

Nashville TN 37232

### Competing of Interest – COPE

Oluwole reports: Consultancy and advisory board for Pfizer, Kite, Gilead, AbbVie, Janssen, TGR Therapeutics, ADC, Novartis, Epizyme, Curio Science, Nektar, Cargo, Caribou, Autolus, Bioheng, and Allogene.

Institution funding: Kite, Pfizer, Daichi Sankyo, Cargo, Caribou, Sana, Century Allogene.

Honoraria: Pfizer, Gilead, ADC

Dholaria reports: Institutional research funding: Janssen, Angiocrine, Pfizer, Poseida, MEI, Orcabio, Wugen, Allovirm Adicet, BMS, Molecular templat. Consultancy/Advisor: MJH BioScience, Janssen, ADC therapeutics, Gilead/Kite, Autotus, Poseida, Accrotech

Savani: No COI

Bhaskar: No COI
